# A Chinese Herbal Formula Suppresses Colorectal Cancer Migration and Vasculogenic Mimicry Through ROS/HIF-1*α*/MMP2 Pathway in Hypoxic Microenvironment

**DOI:** 10.3389/fphar.2020.00705

**Published:** 2020-05-15

**Authors:** Shaoqi Zong, Yufei Tang, Wen Li, Susu Han, Qi Shi, Xiaofeng Ruan, Fenggang Hou

**Affiliations:** ^1^Department of Oncology, Shanghai Municipal Hospital of Traditional Chinese Medicine, Shanghai University of Traditional Chinese Medicine, Shanghai, China; ^2^Graduate School of Shanghai University of Traditional Chinese Medicine, Shanghai, China; ^3^Department of Dermatology, Shuguang Hospital Affiliated to Shanghai University of Traditional Chinese Medicine, Shanghai, China

**Keywords:** Astragalus Atractylodes mixture, colorectal cancer, migration, vasculogenic mimicry, hypoxia

## Abstract

Various malignant tumors, including colorectal cancer, have the ability to form functional blood vessels for tumor growth and metastasis. Vasculogenic mimicry (VM) refers to the ability of highly invasive tumor cells to link each other to form vessels, which is associated with poor cancer prognosis. However, the antitumor VM agents are still lacking in the clinic. Astragalus Atractylodes mixture (AAM), a traditional Chinese medicine, has shown to inhibit VM formation; however the exact mechanism is not completely clarified. In this study, we found that HCT-116 and LoVo could form a VM network. Additionally, hypoxia increases the intracellular reactive oxygen species (ROS) level and accelerates migration, VM formation in colorectal cancer cells, while N-Acetylcysteine (NAC) could reverse these phenomena. Notably, further mechanical exploration confirmed that the matrix metalloprotease 2 (MMP2) induction is ROS dependent under hypoxic condition. On the basis, we found that AAM could effectively inhibit hypoxia-induced ROS generation, migration, VM formation as well as HIF-1*α* and MMP2 expression. *In vivo*, AAM significantly inhibits metastasis of colorectal cancer in murine lung-metastasis model. Taken together, these results verified that AAM effectively inhibits migration and VM formation by suppressing ROS/HIF-1*α*/MMP2 pathway in colorectal cancer under hypoxic condition, suggesting AAM could serve as a therapeutic agent to inhibit VM formation in human colorectal cancer.

## Highlights

1. High level of ROS is associated with migration in colorectal cancer cells.
2. In highly metastatic colorectal cancer cells, HCT-116 and lovo could form vasculogenic mimicry.
3. Up-regulation of MMP2 under hypoxia requires ROS generation in colorectal cancer cells.
4. AAM effectively inhibits migration and VM formation by suppressing ROS/HIF-1*α*/MMP2 axis under hypoxic condition.

## Introduction

Colorectal cancer (CRC) is the third most common cancer and third cause of death in cancer patients worldwide, and the incidence rate is increased in males of the 40–79 age group (Siegel et al., 2018). Tumor invasion and metastasis are the main cause of death in cancer patients (Sun et al., 2007b). In traditional opinion, angiogenesis is the main reason for tumor metastasis, therefore, antiangiogenesis drugs were developed and used for treating malignant tumors, including CRC (Ghiringhelli et al., 2014). However, these agents may cause hypoxia, then promotes tumor progression, treatment resistance, and metastasis (Jain, 2014). Additionally, tumor hypoxia has been demonstrated to promote the formation of vasculogenic mimicry (VM) (Comito et al., 2011; Du et al., 2014; Li et al., 2016; Wang et al., 2017) VM consists of tumor cells, which link each other and form cell extracellular matrix-rich networks [peridiodic acid-Schiff (PAS)-positive]; studies have demonstrated that tumors, including CRC, may develop VM (Liu et al., 2012; Su et al., 2016), which is associated with poor prognosis and is partly responsible for resistance to anti-angiogenic agents (Liu et al., 2013; Yang et al., 2016).

VM formation is induced by many factors. Recent studies revealed that mild hypoxia (1–3% O_2_) increases mitochondrial ROS production (Guzy et al., 2005; Klimova and Chandel, 2008). ROS, in turn, promotes ferrous ion (Fe^2+^) oxidation then inhibits the activation of prolyl hydroxylases (PHDs) and hence stabilizes HIF-alpha (Guzy et al., 2005; Lu et al., 2005). Additionally, it has been demonstrated that antioxidants as vitamin C and N-acetylcysteine (NAC) could inhibit HIF-1*α* stabilization then diminishes tumorigenesis in MYC-dependent murine models (Gao et al., 2007). Moreover, HIF-1*α* overexpression was reported as a poor prognosis factor for CRC patients (Kwon et al., 2010).

Although these studies suggest that hypoxia and ROS/HIF-1*α* signaling play important roles in CRC progression, the mechanism of hypoxia mediated CRC progression is not well understood. Furthermore, previous studies confirmed that antiangiogenic agents did not significantly inhibit the formation of VM, and they even induced extracellular matrix-rich tubular network formation (van der Schaft et al., 2004). Thus, it is very urgent to probe the mechanism of vasculogenic mimicry and develop related antivascular drugs that specifically target VM.

It has been confirmed that Traditional Chinese Medicine (TCM) could effectively prolong survival time, reduce side effect, enhance treatment effect in cancer patients (Ling et al., 2014; Nie et al., 2016; Liao et al., 2017). Notably, many researchers found that TCM has a potential value in preventing recurrence and metastasis in CRC patients combined with western medicine therapy (Yang et al., 2008; Shi et al., 2017). Astragalus Atractylodes mixture (AAM) was constructed according to traditional Chinese medicine theories and clinical experience, which consisted of *Astragalus membranaceus Fisch. ex Bunge*. (AMF), *Atractylodes macrocephala Koidz*. (AMK), *Actinidia arguta (Siebold & Zucc.) Planch. ex Miq*. (AAP), *Curcuma aromatica Salisb*. (CAS), *Benincasa hispida (Thunb.) Cogn*. (BHC), and *Ficus pumila L*. (FPL). Many of these herbs have antitumor and antioxidant effect. Astragaloside IV (Astragalus membranaceus extract) suppresses tumor progression through inhibiting Akt/GSK-3*β*/*β*-catenin signaling axis and enhances chemosensitivity *via* targeting caveolin-1 (CAV-1), which led to oxidative stress (Jia et al., 2018; Zheng et al., 2018). *Actinidia arguta* exerts antitumor effect by inhibiting tumor cell proliferation (Lim et al., 2016), Additionally, oleanolic acid (*Actinidia arguta (Siebold & Zucc.) Planch. ex Miq*. extraction) has antioxidant activity and attenuates tumor progression *via* inhibiting epithelial–mesenchymal transition (EMT) (Bai et al., 2018; Wang et al., 2018). Moreover, our previous study found that AAM could effectively prevent CRC progression *via* inhibiting tumor angiogenesis and vasculogenic mimicry (VM) in murine model (Manman Yu et al., 2011; Hou et al., 2016). However, the details of anti-VM mechanisms of AAM are not well understood until now.

Based on the theory that hypoxia promotes tumor progression *via* activating ROS/HIF-1*α* signaling axis, we speculate that AAM might exert antitumor effect through eliminating ROS production in a tumor hypoxia microenvironment. In this report, the effect of AAM on hypoxia-induced vasculogenic mimicry (VM) was investigated *in vitro*. Moreover, murine lung experimental metastasis model was applied to investigate whether AAM inhibits tumor metastasis. The results showed that AAM suppresses CRC vasculogenic mimicry (VM) formation and migration through inhibiting the ROS/HIF-1*α* pathway in a hypoxic microenvironment. The results of this study may explain the potential antitumor mechanisms of AAM and provide the basis for the clinical treatment of CRC.

## Materials and Methods

### Reagents and Antibodies

Reagents were purchased as follows: AAM was purchased from Jiangyin Tianjiang Pharmaceutical Co., Ltd (Jiangsu, China) which has evaluated the quality according to Chinese Pharmacopoeia (CP) (2015); the herb granules are shown in **Table 1**. Dulbecco’s modified Eagle’s medium (DMEM) medium (#KGM12800, Keygen, Nanjing, China), RPMI-1640 medium (#KGM31800, Keygen, Nanjing, China), Fetal Bovine Serum (#100991141, Gibco Life Technologies, Australia), Matrigel matrix (#356234, BD Bioscience, USA), NAC (#A725, Sigma-Aldrich, USA), ROS assay kit (#E004, Nanjing JianCheng Bioengineering Institute), IP lysis buffer (#P0013, Beyotime Biotechnology), BCA protein Assay kit (#P0010, Beyotime Biotechnology), CCK-8 assay kit (#KGA317s, Keygen Biotech, Jiangsu, China), Trizol reagent (sigma, T9424), PrimeScript™ RT Master Mix (##RR036A, Takara, Japan), SYBR Premix Ex Taq (Takara, RR420A, Japan). Antibodies were used as follows: the primary antibodies used for western blot: MMP2 (EPR1184) Rabbit mAb (#ab92536, Abcam), VE-Cadherin Rabbit mAb (EPR18229) (#ab205336, Abcam), Eph-receptor A2 (RM-0051-8F21) Rabbit mAb (#ab73254, Abcam), MMP9 Rabbit pAb (#A0289, ABclonal), HIF-1alpha (H1alpha67) Mouse mAb (NB100-105, NOVUS), Immobilon western chemilum HRP substrate (ECL, #KGP1123, KeyGen).

**Table 1:** The compositions of Astragalus Atractylodes mixture (AAM).

Chinese name	Latin name	Taxonomic names	Weight(g)	Lot number	Part used
Huang qi	*Astragali radix*	Astragalus membranaceus Fisch. ex Bunge.	20	18102061	Root
E zhu	*Curcumae radix*	Curcuma aromatica Salisb.	10	18111421	rhizome
Bai zhu	*Atractylodis macrocephalae rhizoma*	Atractylodes macrocephala Koidz.	30	18121121	rhizome
Dong gua zi	*Benincasae semen*	Benincasa hispida (Thunb.) Cogn.	10	18061121	seed
Teng li gen	*Actinidia arguta*	Actinidia arguta (Siebold & Zucc.) Planch. ex Miq.	30	18061101	Root
Bi li guo	*Ficus pumila linn*	Ficus pumila L.	15	18082011	fruit

### Cell Culture and Treatment

The human colorectal cancer cell lines HCT-116 and LoVo were purchased from Shanghai Institutes for Biological Sciences (SIBS). HCT116 cells were cultured in DMEM medium, and LoVo cells were incubated in RPMI-1640 medium. All the cell culture medium was supplemented with 10% FBS, 10% penicillin, and streptomycin (100 U/ml). The normoxia cells were placed in 5% CO_2_ and 21% O_2_ cell incubator; hypoxia model was performed in three gas chambers (HF100) (5% CO_2_, 1% O_2_, 94% N_2_). Colorectal cancer cells were seeded in a 6-well plate; when the cell fusion reached about 30%, which were starved for 12 h then treated with 10 mM NAC, with or without AAM (2.5, 5, and 10 mg/ml) for 24 h, cells treated with DMEM medium were used as negative control. Finally, the treated cells were collected for further analysis as described below.

### Preparation of Astragalus Atractylodes Mixture

The herb granules, *Astragalus membranaceus Fisch. ex Bunge* (AMF), Atractylodes macrocephala Koidz. (AMK), Actinidia arguta (Siebold & Zucc.) Planch. ex Miq. (AAP), Curcuma aromatica Salisb. (CAS), Benincasa hispida (Thunb.) Cogn. (BHC), and Ficus pumila L. (FPL) were mixed at a 4:6:3:2:2:3 ratio (**Table 1**). Then the herb granules were extracted with 100 ml of ethanol for three times. The extracts were combined and dissolved in 1 ml of methanol. After centrifugation at 1,000 rpm for 5 min, the supernatant was collected and filtrated through a 0.2 μm membrane filter and stored in −20°C before use. The herb granules were purchased from Jiangyin Tianjiang Pharmaceutical Co., Ltd (Jiangsu, China) which has evaluated the quality according to Chinese Pharmacopoeia (CP) (2015).

### Extraction and HPLC/MS Analysis

The herb granules, *Astragalus membranaceus Fisch. ex Bunge* (AMF), Atractylodes macrocephala Koidz. (AMK), Actinidia arguta (Siebold & Zucc.) Planch. ex Miq. (AAP), Curcuma aromatica Salisb. (CAS), Benincasa hispida (Thunb.) Cogn. (BHC), and Ficus pumila L. (FPL) were mixed at a 4:6:3:2:2:3 ratio and were extracted with 100 ml of ethanol for three times. The extracts were combined and dissolved in 1 ml of methanol. After centrifugation, the supernatant was used for HPLC Q-TOF/MS analyses. The HPLC/MS analyses were carried out on an Agilent 6538 UHD Accurate-Mass Q-TOF LC/MS (Agilent Technologies Inc., USA) with a XSelect HSS T3 C18 column (2.1 × 100 mm, 2.5 μm) eluting with a flow rate of 0.4 ml·min^−1^ over a 19 min gradient as follows: T = 0 min, 5% B; T = 2 min, 5% B; T = 15 min, 95% B; T = 19 min, 95% B (phase A, H_2_O with 0.1% HCOOH; phase B, CH_3_CN with 0.1% HCOOH).

### Assay of Intracellular ROS

A ROS assay kit was used to determine intracellular ROS accumulation. Ten minutes before the end of treatments, cells were washed with PBS then treated with 10 μM DCFH-DA at room temperature for 30 min in the dark, then the cells were harvested, washed, and responded in PBS solution at a density of 5 × 10^6^ cells/ml. The cells were incubated with FITC-conjugated DCF fluorescence then analyzed using FACSAria III flow cytometer. The DCF fluorescence intensity was detected, qualified, and defined as intracellular ROS level.

### Cell Viability Assay

The cell viability was measured by CCK-8 assay. Briefly, three groups were conducted; the negative control group was conducted with no cells, the experimental group was conducted with different concentrations (0, 0.5, 1, 2, 4, 6, 8, 12, 16 mg/ml) of AAM, and the positive control was conducted with no drugs. The cells were placed in 96-well plates in a density of 8 × 10^3^ cells/well. After 6 h, cells were incubated with AAM at different concentrations for 24 h. Then the cells were incubated with 10% CCK-8 cell culture medium for 1 h at 37°C. the absorbance of optical density (OD) was measured in a microplate reader at 450 nm (Thermo scientific, Waltham, MA, USA). Cell viability was expressed as a percentage of the control according to the manufacturer’s instruction.

### Wound Healing Assay

In wound healing assay, cells were plated in 24-well culture plates. After the cells formed a cell monolayer and serum starvation for 12 h, a 20 μl pipet tip was used to scratch a straight line in the center of the well, then cells were treated with medium alone, medium with AAM (2.5 mg/ml), or NAC (10 mM), hypoxia medium, hypoxia medium with AAM (2.5 mg/ml), or NAC (10 mM) for 24 h. To measure the cells’ migration ability, the same area of the well was observed in 0 and 24 h after scratching. The cells’ scratch area was measured by Image J.

### Transwell Migration Assay

Additionally, the cell migration was measured with Transwell system (#3422, Corning). Firstly, 5 × 10^4^ cells in 200 μl were loaded in the upper chamber in serum-free medium supplemented with 2.5 mg/ml AAM or 10 mM NAC. Then the chambers were placed into 24-well culture plates which contained 500 μl DMEM/1640 medium with 15% serum. After 24 h of incubation at 37°C under normoxic and hypoxic conditions, the nonmigrating cells were gently removed using cotton swabs, and then the Transwell membrane containing migrated cells was fixed in 95% ethanol for 10 min and stained by 0.1% crystal violet. The number of migrated cells was observed using inverted optical microscope.

### VM Formation Assay

In the VM formation assay, 96-well plate was coated with 50 μl Matrigel and allowed to gel for 30 min at room temperature; then cells (2 × 10^4^ cells per well) were plated on the surface of Matrigel-coated plates and incubated under hypoxic or normoxic conditions. After 24 h, three random fields of each well were taken using inverted optical microscope (Leica DFC280). The number of tube networks was measured using Image J.

### Western Blotting Analysis

Western blot analysis was performed as previously described (Li et al., 2016). Briefly, cells were lysed using western and IP lysis buffer at 4°C for 30 min and then centrifuged at 12,000 g for 10 min. The supernatant was collected, and the total protein concentration was determined using BCA protein Assay kit. The protein lysates were separated by relative concentrations (8, 10, 12%) of SDS polyacrylamide gels, and then the protein electrophoresis equipment (Bio-Rad, Shanghai, China) was used to transfer the proteins onto the nitrocellulose (NC) membrane. The membranes were blocked with 5% nonfat milk in PBST and then incubated with relative primary antibodies (VE-cadherin, EphA2, HIF-1*α*, MMP2 and MMP9) for 12 h at 4°C. The membranes were washed and incubated with horseradish peroxidase (HRP)-conjugated antimouse or antirabbit IgG for 1 h at 37°C. The protein bands were visualized using ECL.

### Quantitative Real-Time PCR

Total cellular RNA was extracted using Trizol reagent according to the manufacturer’s instruction. Reversed transcription was done using PrimerScript™ RT reagent kit (#RR037A, Takara), and quantitative PCR was performed using ABI 7500 sequence Detector; the primer sequences were listed in **Table 2**. Relative mRNA expression levels were analyzed using the 2^−ΔΔCt^ method.

**Table 2:** Primer sequences used in real-time PCR experiment.

Gene name	Primer sequence (5′ 3′)
HIF-1*α*	Forward: CATCTCCATCTCCTACCCACAT
	Reverse: ACTCCTTTTCCTGCTCTGTTTG
	
MMP2	Forward: ATGACAGCTGCACCACTGAG
	Reverse: AGTTCCCACCAACAGTGGAC
	
*β*-actin	Forward: AGCGAGCATCCCCCAAAGTT
	Reverse: GGGCACGAAGGCTCATCATT

### Murine Lung-Metastasis Model

Male 6–8-week-old athymic nude mice (n = 15) were purchased from Shanghai Silaike Experiment Animal Co., Ltd (Shanghai, China). Animals were maintained under specific pathogen-free (SPF) conditions. All animal experimental protocols and animal care procedures were approved by the Medicine Animal Ethics Committee of Shanghai University of Traditional Chinese Medicine. All mice were injected with 2 × 10^6^ HCT-116 cells resuspended in 0.2 ml of PBS in the lateral tail vein. The mice were then randomly divided into three treatment groups: control (normal saline, 13.5 ml/kg, i.g.), N-Acetylcysteine (150 mg/kg/d, i.p.), and AAM (16 mg/g, i.g.). The mice were monitored every day and sacrificed after 50 days. Lungs were inspected for metastatic nodules. Organs were fixed overnight at 4°C in formalin for histological analysis. The number of metastatic tumor nodules on the surface of lungs was counted to assess the extent of metastasis.

### HE Staining

Mice lung specimens were fixed with formalin and embedded with paraffin and then prepared in 5-mm-thick tissue sections. Firstly, hematoxylin was used to stain the sections for 5 min, and the sections were washed with fresh water for 6 min then stained in eosin for 30 s. Five slides of each group were randomly selected and then observed using an inverted phase microscope. Finally, a collaborator who was blinded from the study analyzed the slides results.

### Statistical Analysis

Data are expressed as mean ± standard deviation (SD). Statistical analyses of all data were performed with SPSS 18.0 (IBM Corporation, USA). If the data passed the tests for normality and homogeneity of variance, the one-way ANOVA was selected for statistical analysis, otherwise, a nonparametric test was applied. *P* value of ≤0.05 was considered statistically significant.

## Results

### HPLC Analysis of AAM Extract

**Figure 1:**
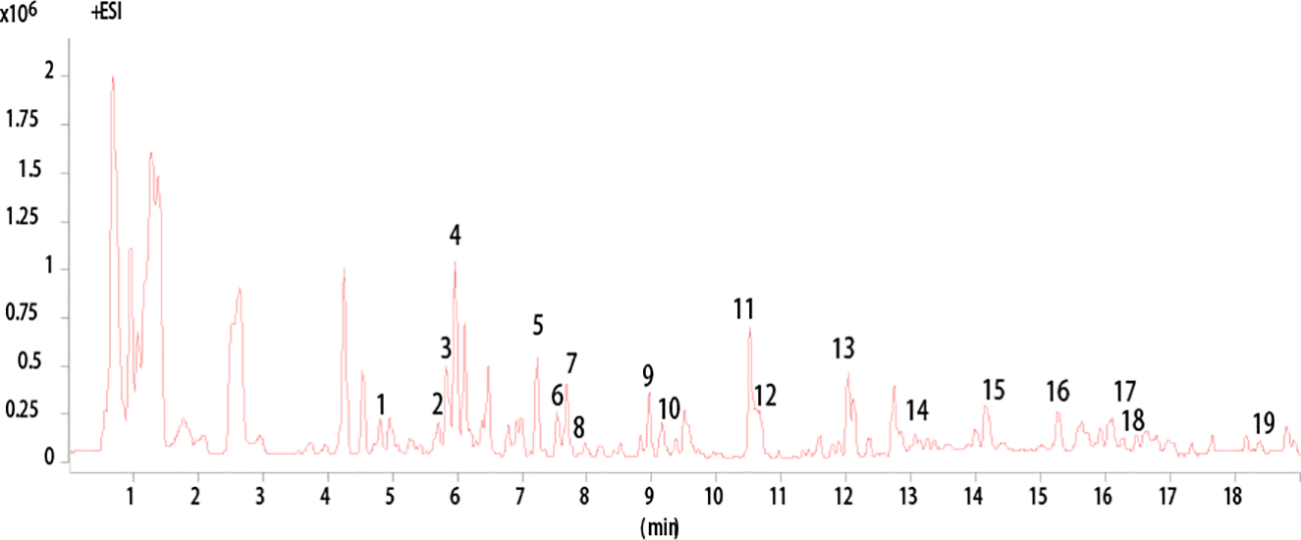
HPLC analysis of AAM extract. Base Peak chromatogram from HPLC Q-TOF/MS analysis of the ethanol extract of AAM (positive ions).

High-performance liquid chromatography Q-TOF mass spectrometry has been applied to analyze the chemical composition of the ethanol extract of AAM (**Figure 1**). Nineteen compounds were deduced as chlorogenic acid, isocurcumenol, rutin, calycosin-7-*O*-*β*-D-glucoside, 4′-methoxyisoflavone-7-*O*-*β*-D-glucoside, (6*R*,11*R*)-3-hydroxy-9,10-dimethoxypterocarpan, luteolin, emodin-3-methyl ether, 5-*O*-caffeoyl quinic acid methyl ester, 3-hydroxy-9,10-dimethoxyptercarpan, curcumenol, atractylenolide III, 2-atractylenolide, isorhamnetin, astraisoflavanglucoside-6″-*O*-malonate, ononin, 3*β*-*O*-acetyl ursolic acid, 3*β*-*O*-acetyl oleanolic acid, and *seco* isolariciresinol 9-*O*-*β*-D-glucopyranoside by comparing the fragment ions with those of characteristic components of the herbal preparation reported in literatures (Lin et al., 2000; Doshi et al., 2016; Sun et al., 2017; Zhu et al., 2018) (**Table 3**).

**Table 3:** HPLC Q-TOF/MS analysis results for chemical contents in the ethanol extract of AAM (positive ions).

Peak no.	t*_R_* (min)	Compounds name	Formula	Calcd. *m*/*z*	Experimental *m*/*z*	Source
1	4.82	Chlorogenic acid	C_16_H_18_O_9_	355.1029	355.1041	FPL
2	5.84	Isocurcumenol	C_15_H_22_O_2_	235.1698	235.1715	CAS
3	5.93	Rutin	C_27_H_30_O_16_	611.1612	611.1623	BHC
4	5.97	Calycosin-7-*O*-*β*-D-glucoside	C_22_H_22_O_10_	447.1291	447.1330	AMF
5	7.25	4′-Methoxyisoflavone-7-O-β-D-glucoside	C_23_H_26_O_8_	431.1706	431.1384	AMF
6	7.55	(6*R*,11*R*)-3-Hydroxy-9,10-dimethoxypterocarpan	C_16_H_12_O_6_	301.0712	301.1093	AMF
7	7.61	Luteolin	C_15_H_10_O_6_	287.0556	287.1252	BHC
8	7.69	Emodin-3-methyl ether	C_16_H_12_O_5_	285.0763	285.0784	AAP
9	8.84	5-*O*-Caffeoyl quinic acid methyl ester	C_17_H_20_O_8_	353.1236	353.2303	FPL
10	9.37	3-Hydroxy-9,10-dimethoxyptercarpan	C_17_H_16_O_5_	301.1076	301.1072	AMF
11	10.52	Curcumenol	C_15_H_22_O_2_	235.1698	235.1719	CAS
12	10.69	Atractylenolide III	C_15_H_20_O_3_	249.1491	249.1500	AMR
13	12.03	2-Atractylenolide	C_15_H_20_O_2_	233.1542	233.1561	AMR
14	13.14	Isorhamnetin	C_16_H_12_O_7_	317.0661	317.2098	BHC
15	14.39	Astraisoflavanglucoside-6′′-*O*-malonate	C_26_H_30_O_13_	551.1765	551.3359	AMF
16	15.32	Ononin	C_22_H_22_O_9_	431.1342	431.3156	AMF
17	16.48	3*β*-*O*-Acetyl ursolic acid	C_32_H_50_O_4_	499.3787	499.3794	AAP
18	16.65	3*β*-*O*-Acetyl oleanolic acid	C_32_H_50_O_4_	499.3787	499.3789	AAP
19	18.39	*seco* Isolariciresinol 9-*O*-*β*-D-glucopyranoside	C_26_H_34_O_11_	523.2179	523.3768	FPL

AMF, Astragalus membranaceus Fisch. ex Bunge; CAS, Curcuma aromatica Salisb; AMK, Atractylodes macrocephala Koidz; BHC, Benincasa hispida (Thunb.) Cogn; AAP, Actinidia arguta (Siebold & Zucc.) Planch. ex Miq; FPL, Ficus pumila L.

### NAC Inhibit Colorectal Cancer Cell Migration Under Hypoxia Condition

**Figure 2:**
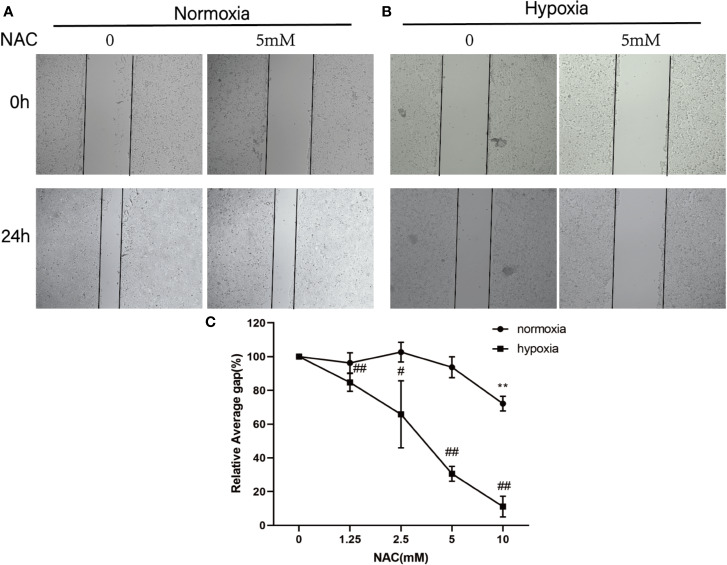
NAC inhibits colorectal cancer cell migration under hypoxia condition. Wound-healing assay for evaluating the migration ability of HCT-116 cells under normoxia **(A)** and hypoxia condition **(B)**; cells were photographed at 0 and 24 h under 50× magnification. **(C)** The relative scratch area was measured by Image J (normoxia group, **P < 0.01; hypoxia group, ^#^*P* < 0.05, ^##^*P* < 0.01, ^###^*P* < 0.001).

To investigate whether ROS affects the migration of colorectal cancer, HCT-116 cells were treated with different concentrations (0, 1.25, 2.5, 5, 10 mM) of NAC and then exposed in normoxia and hypoxia conditions. The migration ability of HCT-116 cells was evaluated using wound-healing assay. As shown in the **Figure 2**, the migration distance of HCT-116 cells is dose dependent on hypoxia condition; however, the migration ability is not affected by the low dose (1.25, 2.5, 5 mM) NAC in normoxia condition. These results suggest that the ROS level regulated the migration ability of colorectal cancer cells under hypoxia condition.

### Hypoxia Increases ROS Level in Colorectal Cancer Cells and NAC Could Reverse This Phenomenon

**Figure 3:**
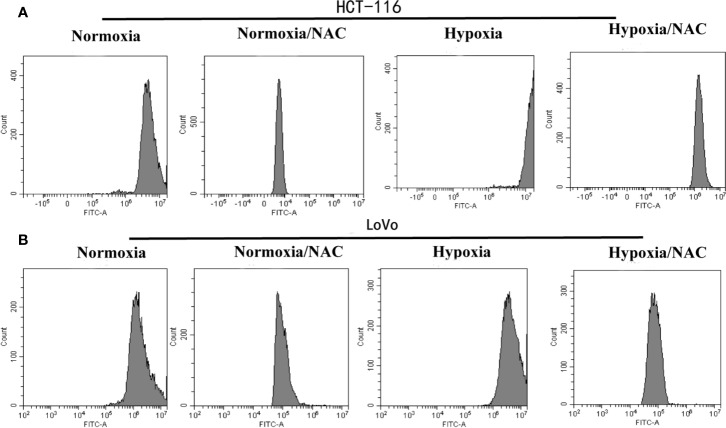
Hypoxia increases the ROS level in colorectal cancer cells and NAC could reverse this phenomenon. The **(A)** HCT-116 and **(B)** LoVo cells were cultured in hypoxic condition (1% O_2_) and treated with NAC for 24 h, then treated with DCFH-DA (10 μM) for 30 min; the DCF fluorescence intensity was measured by flow cytometry.

To determine whether hypoxia increases the intracellular ROS levels in colorectal cancer cells, firstly, the cells that could form VM were selected from highly metastatic human CRC cell lines (DLD-1, SW620, HCT-116, LoVo). The results showed that HCT-116 and LoVo cells could form VM (**Figure S1**). Then, these two cell lines were cultured in hypoxic condition (1% O_2_) for 24 h and then treated with DCFH-DA (10 μM) for 30 min. Finally, the cells were collected to measure the DCF fluorescence intensity using flow cytometry. The results revealed that hypoxia could increase the DCF fluorescence intensity. In addition, the cells that were treated with 10 mM NAC (a known scavenger of ROS) for 24 h could decrease the intracellular ROS level (**Figures 3A, B**). These data showed that hypoxia increases the ROS level in colorectal cancer cells, and NAC could reverse this phenomenon.

### Hypoxia Accelerates Migration and Vasculogenic Mimicry Required ROS Generation

**Figure 4:**
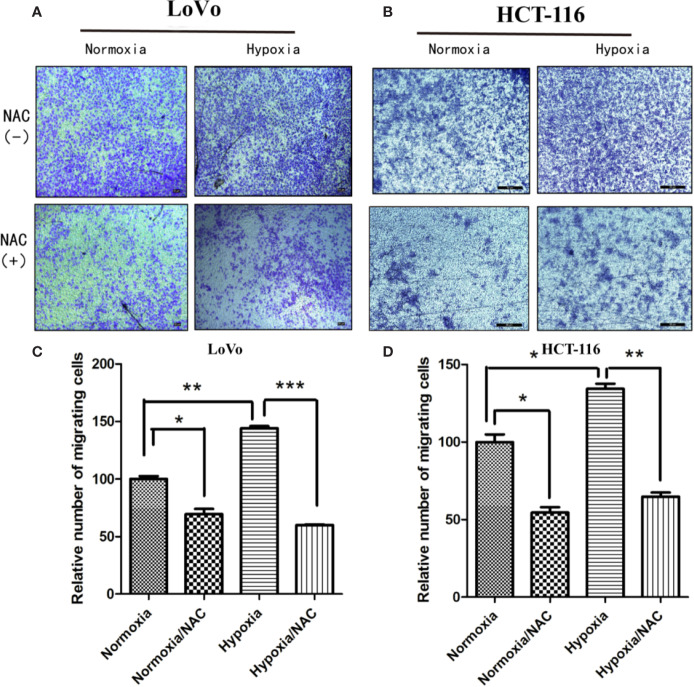
Hypoxia accelerates migration and requires ROS generation. Transwell assay was used to evaluate the effect of hypoxia and ROS on migration ability. LoVo **(A)** and HCT-116 **(B)** cells were cultured in hypoxic condition (1% O_2_) and treated with NAC for 24 h; cells on the lower surface of the Transwell membrane were stained with 0.1% crystal violet; the number of migrated cells was photographed under 50× magnification; Data are expressed as mean ± SD, n=3 **(C, D)**. (*P < 0.05, **P < 0.01, ***P < 0.001).

**Figure 5:**
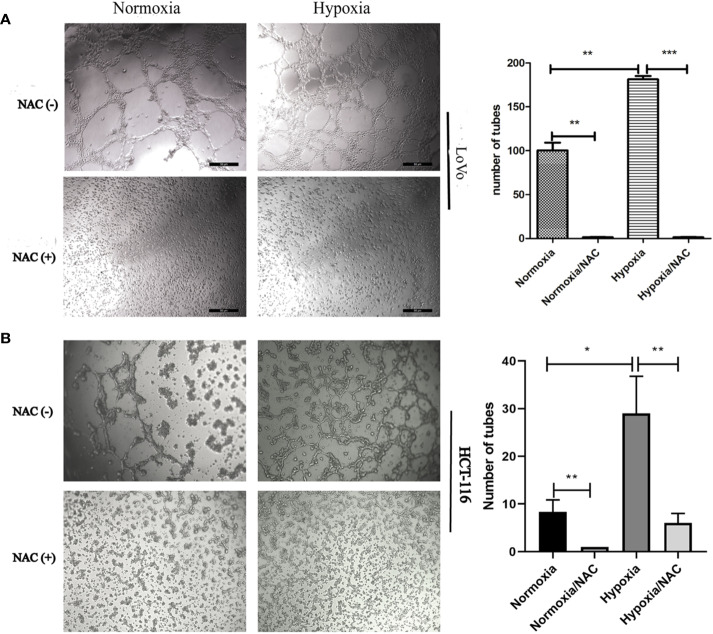
Hypoxia accelerates vasculogenic mimicry and requires ROS generation. Vasculogenic mimicry of LoVo **(A)** and HCT-116 **(B)** cells was assayed after NAC treatment for 36 h at normoxia and hypoxia and photographed under 50× (LoVo) and 50× (HCT-116) magnification; data are represented as mean ± SD from at least three experiments. (*P < 0.05, **P < 0.01, ***P < 0.001).

In view of the fact that tumor cell migration is not only critical to cancer metastasis, but also essential for tumor VM (Sun et al., 2011; Cao et al., 2013), we explored whether hypoxia accelerates migration in colorectal cancer. HCT-116 and LoVo cells were exposed in hypoxic condition (1% O2) for 24 h, and then their migration ability was analyzed using Transwell migration assay and wound-healing assay. As shown in **Figure S1**, hypoxia could accelerate migration of both HCT-116 and LoVo cells compared to the normoxia and NAC groups (cells treated with 10 mM NAC). In line with the results obtained by Transwell migration assay, the wound-healing assay also showed that colorectal cancer cells migrated more rapidly in hypoxia than in normoxia, and NAC was able to reverse this phenomenon (**Figures 4A, B** and **Figure S2**). In addition, exposure to hypoxia could strongly increase the number of vasculogenic mimicry in colorectal cancer cells, and NAC could significantly inhibit the formation of vasculogenic mimicry (**Figure 5**). Together, these results validate that hypoxia accelerates migration, and vasculogenic mimicry requires ROS generation in colorectal cancer cells.

### Up-Regulation of MMP2 Under Hypoxia Requires ROS Generation

**Figure 6:**
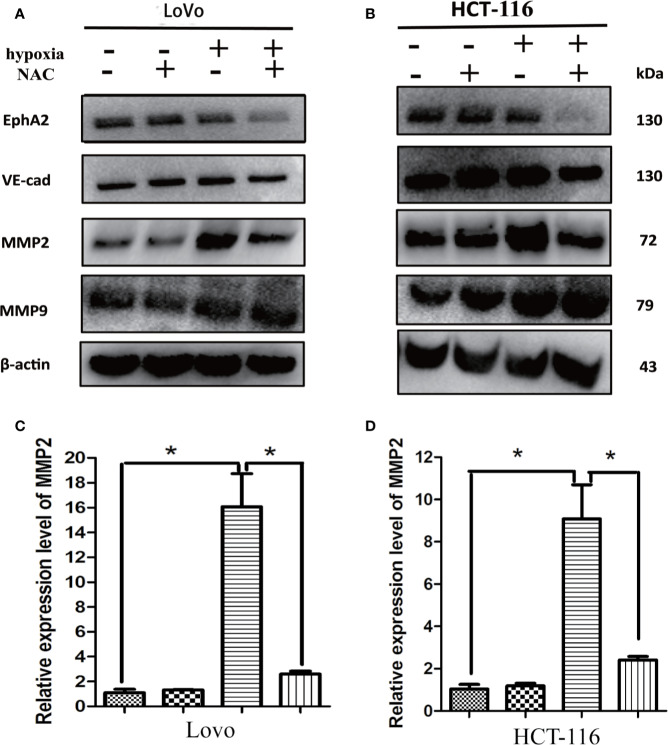
Up-regulation of MMP2 under hypoxia requires ROS generation. **(A, B)** The expressions of EphA2, VE-cad, MMP2, MMP9 using western blotting analysis after the indicated treatment. Results showed that NAC markedly blocked hypoxia-stimulated MMP2 expression compared with the untreated group; **(C, D)** The expressions of MMP2 using RT-PCR after the indicated treatment (*P < 0.05).

It has been found that several promigratory and provasculogenic genes, such as matrix metalloproteinase (MMPs), epithelial cell kinase (EphA2), and vascular endothelial-cadherin (VE-cadherin), play crucial roles in the formation of VM (Hendrix et al., 2001; Seftor et al., 2001; Hess et al., 2003; Sood et al., 2004; Hess et al., 2006; Delgado-Bellido et al., 2017). These genes have been reported as HIF-1*α*-target genes, and ROS is essential for hypoxia-induced HIF-1*α* stabilization (Brunelle et al., 2005; Guzy et al., 2005). To investigate whether hypoxia-induced gene expression is also ROS-dependent, the HCT-116 and LoVo cells were treated with NAC in hypoxia, and then the expression level of MMP2, MMP9, VE-cadherin and EphA2 was detected using western-blot. The results showed that NAC markedly blocked hypoxia-stimulated MMP2 expression compared with the untreated group (**Figures 6A, B**), while the expression levels of other genes, including MMP9, VE-cadherin, and EphA2 were not significantly affected (**Figures 6A, B**). Additionally, RT-PCR results also showed that hypoxia-induced MMP2 expression was suppressed largely by treatment with NAC (**Figures 6C, D**). These data revealed that ROS regulates MMP2 expression in CRC cells under hypoxia.

### AAM Reduces ROS levels and Inhibits Proliferation in Colorectal Cancer Cells

**Figure 7:**
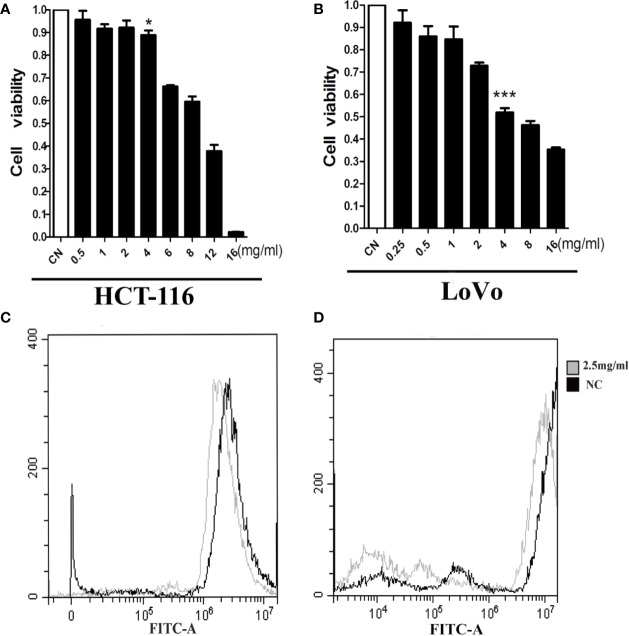
AAM reduces ROS levels and inhibits proliferation in colorectal cancer cells. HCT-116 **(A)** and LoVo **(B)** cell proliferation was assayed at 24 h after treatment with AAM at different concentrations; cells were treated with 2.5 mg/ml AAM, and the ROS production of HCT-116 **(C)** and LoVo **(D)** cells was evaluated using flow cytometry (*P < 0.05, ***P < 0.001).

We assessed the influence of AAM on the proliferation of colorectal cancer cells including HCT-116 and LoVo. Marked inhibition of growth was shown in these two cells at 24 h (**Figures 7A, B**). The 50% inhibitive concentration (IC50) of AAM was calculated. Specifically, the IC50 values of AAM for HCT-116 and LoVo were 10 and 6 mg/ml. In addition, to determine whether AAM could inhibit ROS generation, the cells were treated with 2.5 mg/ml AAM for 24 h, and then the ROS level was detected using flow cytometry. As shown in the **Figures 7C, D**, AAM was able to markedly decrease the intracellular ROS level in colorectal cancer cells. These results indicated that AAM could reduce intracellular ROS levels and inhibit proliferation in colorectal cancer cells.

### AAM Inhibits Migration and VM Formation Under Hypoxia

**Figure 8:**
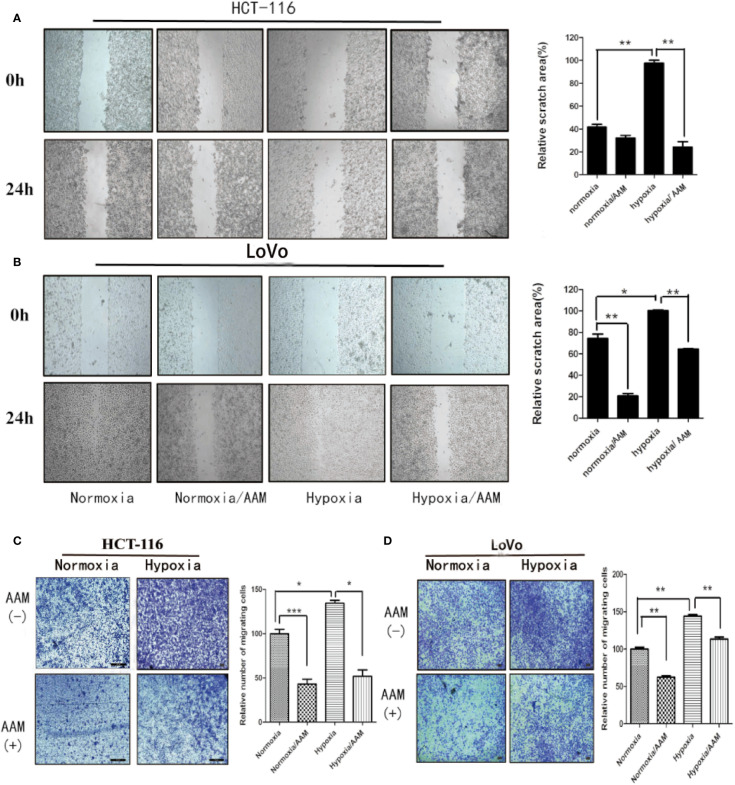
AAM inhibits migration of HCT-116 and LoVo cells. **(A, B)** Wound-healing assays of HCT-116 and LoVo cells. The cells were treated with medium alone, AAM (2.5 mg/ml), hypoxia medium, hypoxia medium with AAM (2.5 mg/ml) for 24 h. The representative images were shown in magnification of 100×. **(C, D)** Transwell assay was used to evaluate the effect of AAM on HCT-116 and LoVo cell migration. Migrated cells were stained and captured by microscope (50×); cell numbers are compared between groups. (*P < 0.05, **P < 0.01, ***P < 0.001).

**Figure 9:**
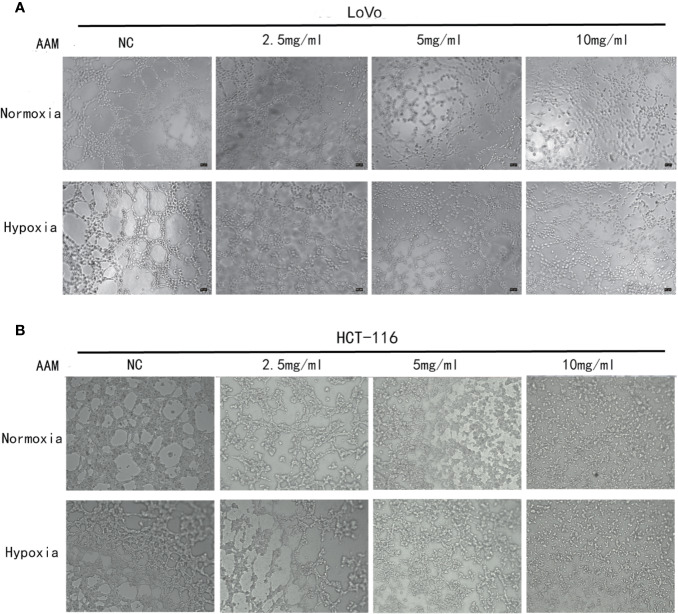
AAM inhibits VM formation of HCT-116 and LoVo cells. VM formation assay. HCT-116 **(A)** or LoVo **(B)** cells were seed into 96-well plate which had been precoated with matrigel and incubated with AAM (2.5, 5, and 10 mg/ml) under normoxia and hypoxia. The representative images were shown in magnification of 50× (HCT-116) and 50× (LoVo).

We investigated the effect of AAM on hypoxia-induced migration and VM formation. The wound-healing assay showed that AAM could significantly suppress the hypoxia-induced migration of colorectal cancer HCT-116 and LoVo cells (**Figures 8A, B**). The Transwell migration assay indicated that the hypoxia-induced migration of HCT-116 and LoVo cells was also inhibited by AAM (**Figures 8C, D**). Furthermore, the VM formation assay showed that AAM strongly inhibited the VM formation in a dose-dependent manner in hypoxia and normoxia, and hypoxia could significantly increase the VM formation when compared to the normoxia group (**Figures 9A, B**). Together, these results validate that AAM could inhibit the hypoxia-induced migration and VM formation.

### AAM Inhibits Hypoxia-Induced VM Formation Involving HIF-1*α* and MMP2

**Figure 10:**
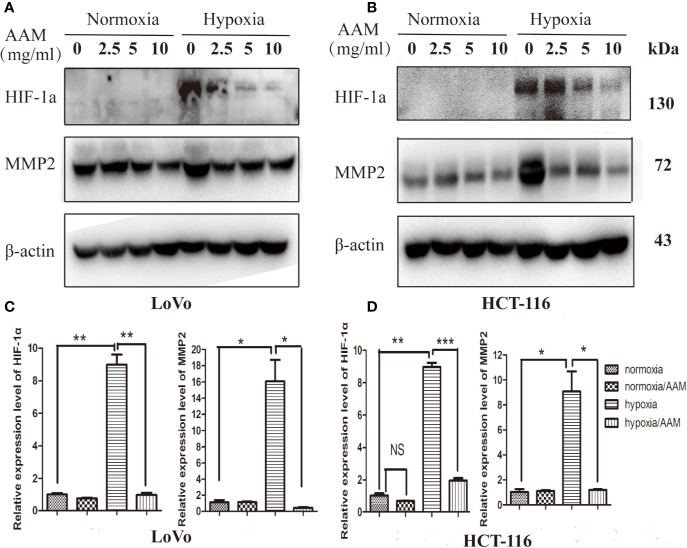
AAM inhibits hypoxia-induced VM formation involving HIF-1*α* and MMP2. The HCT-116 **(A)** and LoVo **(B)** cells were treated with different concentrations of AAM under hypoxia and normoxia; the protein expression of HIF-1*α* and MMP2 was detected by western blotting. **(C, D)** The mRNA expression of HIF-1*α* and MMP2 was measured by RT-PCR (*P < 0.05, **P < 0.01, ***P < 0.001).

As reported above, hypoxia could mediate VM formation *via* the ROS/HIF-1*α*/MMP2 axis. AAM could reduce the hypoxia-induced ROS generation and inhibit VM formation. Therefore, we hypothesize that AAM could reverse this phenomenon *via* the ROS/HIF-1*α*/MMP2 axis under hypoxia. We evaluated the effect of AAM on the expression of HIF-1*α* and MMP2 under normoxia and hypoxia. Western blotting showed that HIF-1*α* and MMP2 protein levels were significantly diminished by AAM in hypoxia (**Figures 10A, B**). RT-PCR showed that AAM also suppressed HIF-1*α* and MMP2 mRNA expression (**Figures 10C, D**). These results revealed that AAM could inhibit hypoxia-induced VM formation involving HIF-1*α* and MMP2.

### AAM Inhibits Metastasis of Colorectal Cancer *In Vivo*

**Figure 11:**
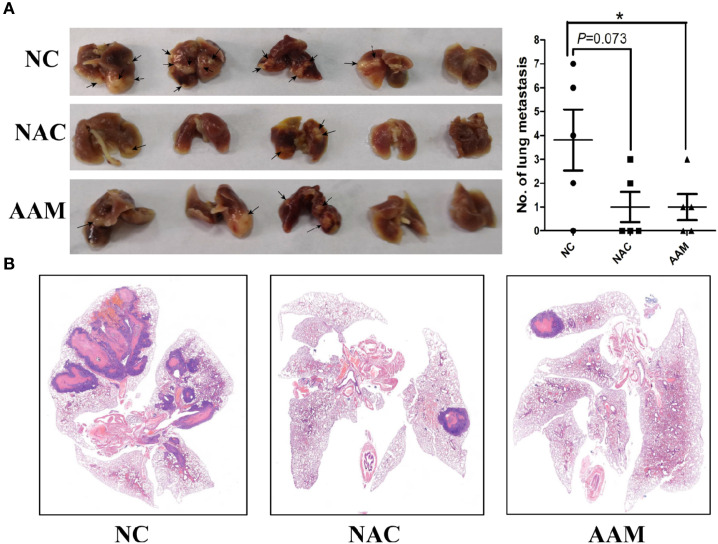
AAM inhibits metastasis of colorectal cancer *in vivo*. **(A)** Representative pictures of lungs of NAC, AAM treated and control groups; the number of metastatic tumor nodules on the surface of the lungs was counted (Right side). **(B)** HE staining was used to determine the metastatic lesions. (*P < 0.05).

We finally evaluated the antitumor effects of AAM in *in vivo* murine lung-metastasis model using NAC as positive control drug. The mice were scarified after tail vein injection for 50 days, and the lung organs were excised. The number of metastatic tumor nodules on the surface of the lungs was counted to assess the extent of metastasis. We found that the number of metastatic tumor nodules tended to be decreased in the NAC group (*P* = 0.073) and significantly decreased in the AAM group (*P* = 0.031) (**Figure 11A**). Then, HE staining was used to determine the metastatic lesions. The results showed that a lot of metastatic lesions were observed in the lung organs with no treatment. However, in mice treated with NAC and AAM, the metastatic lesions decreased markedly (**Figure 11B**).

## Discussion

In 1999, vasculogenic mimicry (VM) was first found by Maniotis (Maniotis et al., 1999) and described as highly invasive melanoma cells that differentiate and obtain endothelial-like characteristic then form vessel-like structures to provide blood supplement for tumor growth, invasion, and metastasis (Hendrix et al., 2003; Sun et al., 2006). Thus, the tumor blood supply is not only dependent on angiogenesis, but is also dependent on VM. Additionally, several studies revealed that antiangiogenic drugs could not significantly inhibit VM formation. Moreover, VM is involved in the development of antiangiogenic agents’ resistance (van der Schaft et al., 2004). Therefore, it is imperative to investigate the relative mechanisms of VM formation and develop target drugs.

Our previous study has demonstrated that VM exists in CRC, and hypoxia-induced EMT promotes VM formation (Li et al., 2016). In this study, we first found colorectal cancer cells HCT-116 and LoVo cells could form VM by screening from four highly invasive CRC cells. As we know, hypoxia is a common feature in solid tumors, which has been reported to mediate a series of tumor biological behaviors, including tumor invasion, epithelial–mesenchymal transition (EMT), angiogenesis, and VM formation (Dusseau and Hutchins, 1988; Sun et al., 2007a; Iwasaki et al., 2018; Liu et al., 2018). Our study also found that hypoxia could promote the migration and VM formation in CRC. However, the mechanism of hypoxia-induced VM is not very clear. Recently, some studies revealed that under hypoxic condition, hypoxia inducible factor 1*α* (HIF-1*α*) promotes VM formation by activating the expression of prometastatic and provasculogenic genes, such as VE-cadherin, EphA2, MMP2, and MMP9 (Hendrix et al., 2001; Hess et al., 2003; Liu et al., 2015; Wang et al., 2017; Duan, 2018). Additionally, mild hypoxia (1–3% O_2_) induced reactive oxygen species (ROS) production then stabilizing HIF-1*α* (Brunelle et al., 2005; Guzy et al., 2005). In the present study, our results showed that hypoxia could increase the ROS production in HCT-116 and LoVo cells, inhibition of ROS production by NAC, the hypoxia-induced VM formation, migration, and MMP2 expression was significantly suppressed. Consequently, we conclude that hypoxia induced VM formation by activating the ROS/HIF-1*α*/MMP2 pathway.

Traditional Chinese Medicine (TCM) has been widely used in China for thousands of years. Currently, several preclinical studies have revealed that TCM can prevent CRC progression and metastasis (Deng et al., 2013; Chen et al., 2017; Shi et al., 2017; Peng et al., 2018). In this study, we examined whether AAM could inhibit hypoxia-induced VM formation and explored relative mechanisms. As reported above, AAM is composed of six kinds of Chinese herbs, including *Astragali radix* (AR), *Curcumae radix Atractylodis macrocephalae rhizoma* (AMR), *Actinidia arguta* (AA), *Curcumae radix* (CR), *Benincasae semen* (BS), and *Ficus pumila linn* (FPL), and a series of studies have reported that some of the compounds and active ingredients of AAM could suppress tumor growth (Lim et al., 2016; Bai et al., 2018; Jia et al., 2018; Zheng et al., 2019). Notably, in these compounds, chlorogenic acid is an antioxidant-rich compound, which could not only effectively scavenge free radicals, but also inhibit cancer-causing pathways (Kavi Rajan and Hussein, 2019). Moreover, it was reported that rutin has effective efficacy in scavenging ROS (Goncalves et al., 2018). Additionally, luteolin was confirmed to have the effect of protecting PC-12 cells from H_2_O_2_ induced injury (Zhang et al., 2019). Therefore, some of the components of AAM could effectively inhibit cancer-causing pathways and scavenge free radicals.

In this study, we found that AAM significantly inhibits proliferation of both HCT-116 and LoVo cells in a dose-dependent manner. Additionally, AAM could inhibit ROS production by flow cytometry analysis. We next examined the anti-VM effect of AAM using VM formation assay; the results suggested that AAM could suppress VM formation in a dose-dependent manner in hypoxia and normoxia conditions. In addition, the tumor cells that migrate to gather each other is an important step in the process of VM formation (Hendrix et al., 2003; Han et al., 2018). Therefore, a drug that effectively inhibits the tumor cell migration would be a potential agent to suppress VM formation. In this context, the results showed that AAM significantly suppressed hypoxia-induced migration of HCT-116 and LoVo cells.

Previous studies have demonstrated that the hypoxia-induced ROS/HIF-1*α* pathway plays important roles in tumor angiogenesis and VM formation. In this study, we found that the up-regulation of MMP_2_ under hypoxia requires ROS generation. Hence, to gain insight into the mechanistic basis for AAM, the effect of AAM on hypoxia-induced ROS/HIF-1α/MMP_2_ pathway was assessed. Western blotting and RT-PCR demonstrated that AAM effectively down-regulated hypoxia-induced HIF-1*α*, MMP_2_ levels. NAC, a known ROS scavenger, has been reported to inhibit tumorigenesis through diminishing HIF-1*α* levels. In the present study, NAC was used as a positive control, and our results proved that NAC could suppress the hypoxia-induced ROS/HIF-1α/MMP_2_ pathway. It also demonstrated AAM to produce an effect similar to NAC in mouse lung-metastasis model. These results reveal that the VM formation inhibitory effect of AAM on CRC depends on the inhibition of the ROS/HIF-1α/MMP_2_ pathway under hypoxic condition.

Nevertheless, our study has some limitations; previous studies reported that focal adhesion kinase (FAK) directly promotes phosphorylation of VE-cadherin in the residue Y658 of VE-cadherin then promoting vasculogenic mimicry (Orsenigo et al., 2012; Delgado-Bellido et al., 2019). Additionally, Xin et al. confirmed that EphA2/FAK/Paxillin pathway contributed to VM formation (Hess et al., 2005; Lu et al., 2013). Furthermore, HIF-1*α* regulates the integrin beta 1 (ITGB1) expression under hypoxia condition (Ju et al., 2017), and ITGB1 promotes the formation of VM through regulating the phosphorylation of Y397 of FAK (Carbonell et al., 2013). Therefore, we will deeply explore the potential molecular mechanism of inhibiting VM formation by AAM in subsequent studies.

## Conclusions

Taken together, these results verified that AAM effectively inhibits migration and VM formation by suppressing the ROS/HIF-1α/MMP2 pathway in colorectal cancer under hypoxic condition, suggesting AAM could serve as a therapeutic agent to inhibit VM formation in human colorectal cancer.

## Data Availability Statement

The datasets generated for this study are available on request to the corresponding author.

## Ethics Statement

The animal study was reviewed and approved by Medicine Animal Ethics Committee of Shanghai University of Traditional Chinese Medicine.

## Author Contributions

FH and SZ conceived and supervised the study. SZ conducted the experiments’ design. YT contributed to the drug preparation. SZ, WL, and YT carried out cell culture and molecular biology experiments. SZ and QS conducted flow cytometry analysis. SH and XR performed the statistical analysis. SZ conducted the manuscript writing; all authors read and approved the final manuscript.

## Funding

This work was supported by the National Natural Science Foundation of China (81473624).

## Conflict of Interest

The authors declare that the research was conducted in the absence of any commercial or financial relationships that could be construed as a potential conflict of interest.

## Supplementary Material

Figure S1 Identification of highly metastatic colorectal cancer cells that could form VM. Highly metastatic CRC cells (DLD-1, SW620, HCT-116 and LoVo cells) were seed into 96 well plate which had been pre-coated with Matrigel for 24 hours. The representative images were shown in magnification of DLD-1, SW620, HCT-116 and LoVo cells (50×).

Figure S2 Hypoxia accelerates migration and requires ROS generation. Wound-healing assays of HCT-116 and LoVo cells. The cells were treated with medium alone, NAC (10mmol/L), hypoxia medium, hypoxia medium with NAC (10mmol/L) for 24 h. The representative images were shown in magnification of 100× (A, B); (C and D) the relative scratch area was measured by Image J (**P* < 0.05).
