# Supplementary figures and images for: A Chinese Herbal Formula Suppresses Colorectal Cancer Migration and Vasculogenic Mimicry Through ROS/HIF-1α/MMP2 Pathway in Hypoxic Microenvironment
Source: Front Pharmacol. 2020 May 15;11:705. doi: 10.3389/fphar.2020.00705 (PMC7242742; doi:10.3389/fphar.2020.00705)

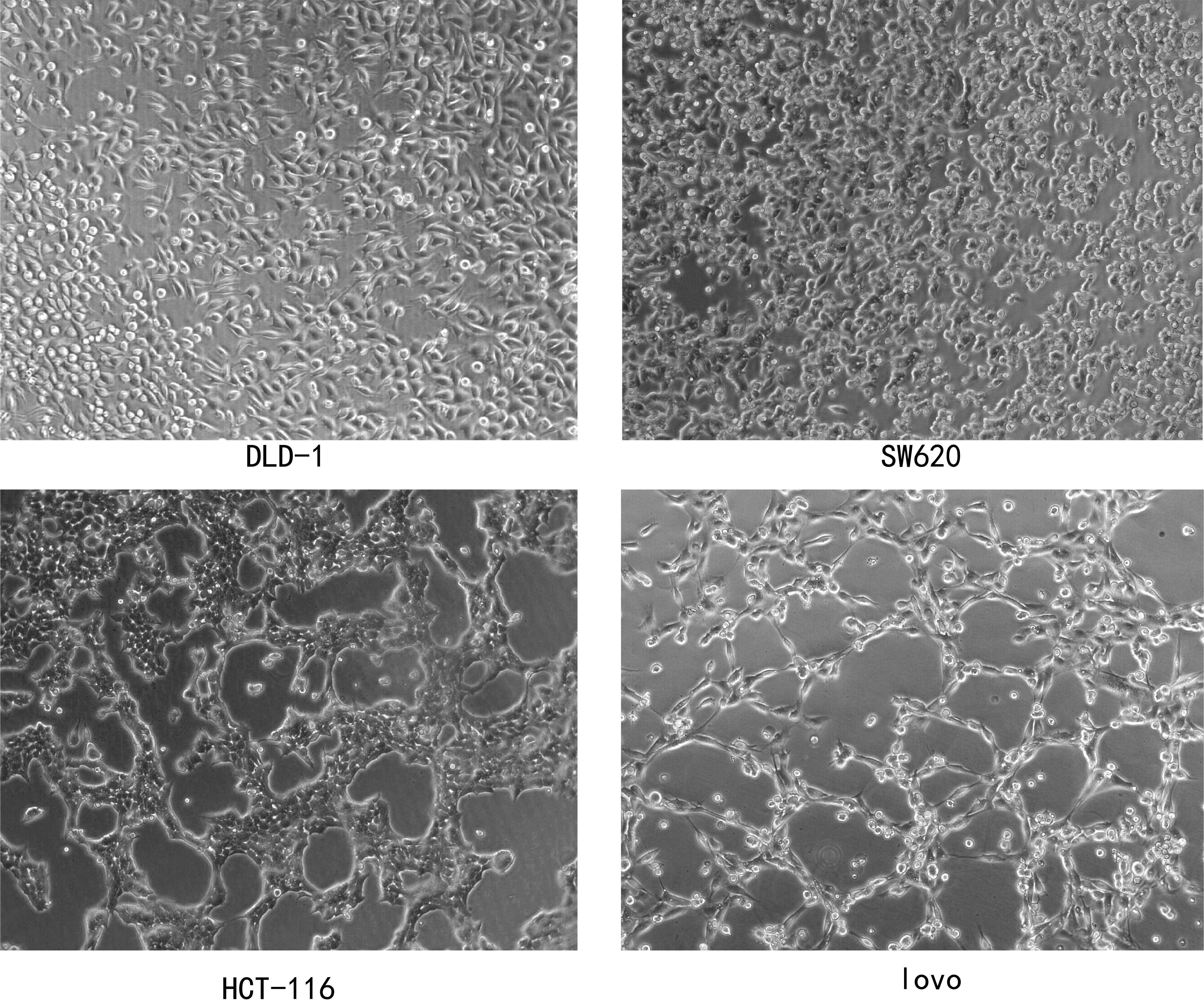

Supplement: Figure S1 — Identification of highly metastatic colorectal cancer cells that could form VM. Highly metastatic CRC cells (DLD-1, SW620, HCT-116 and LoVo cells) were seed into 96 well plate which had been pre-coated with Matrigel for 24 hours. The representative images were shown in magnification of DLD-1, SW620, HCT-116 and LoVo cells (50×). [file Image_1.jpeg]

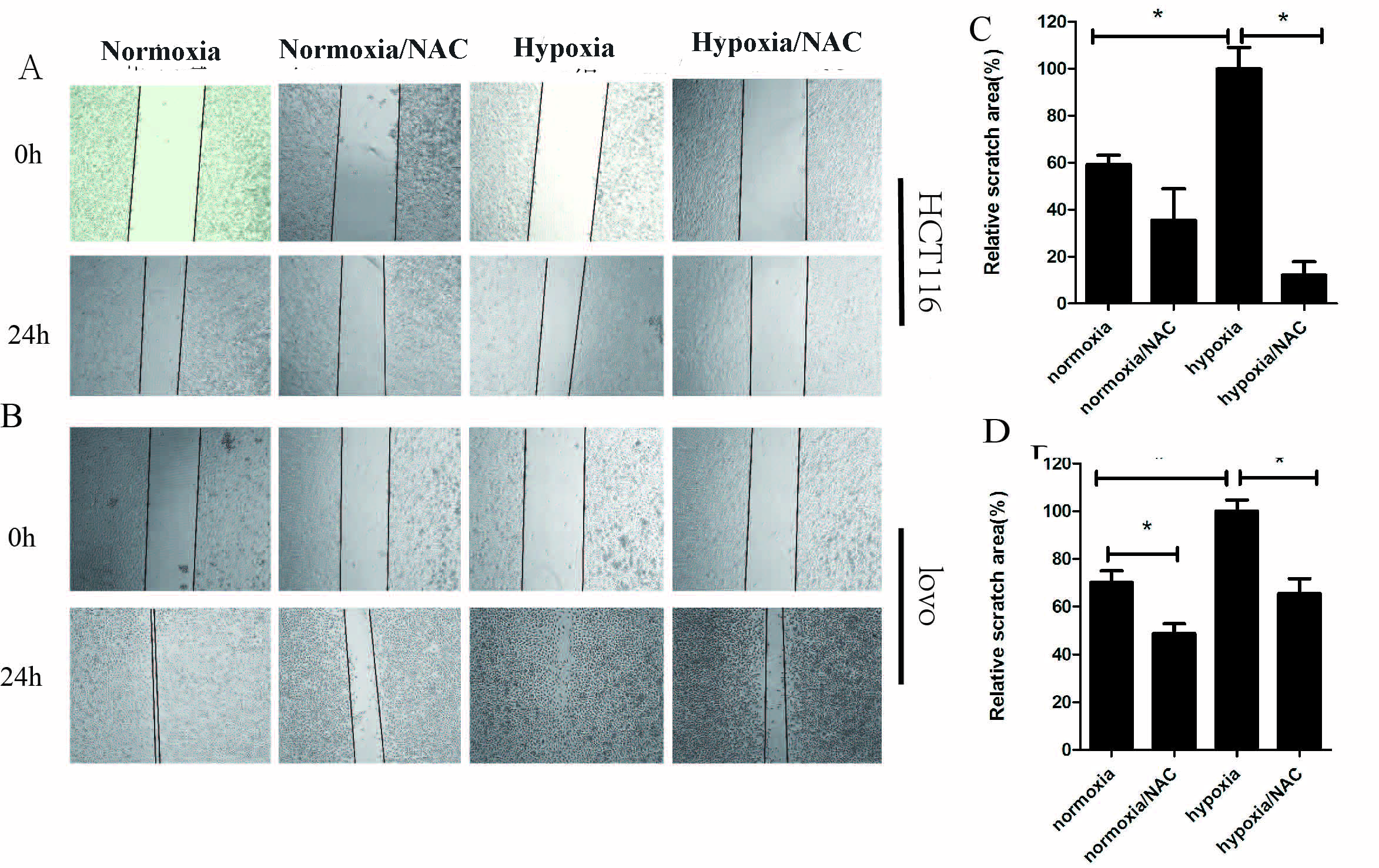

Supplement: Figure S2 — Hypoxia accelerates migration and requires ROS generation. Wound-healing assays of HCT-116 and LoVo cells. The cells were treated with medium alone, NAC (10mmol/L), hypoxia medium, hypoxia medium with NAC (10mmol/L) for 24 h. The representative images were shown in magnification of 100× (A, B); (C and D) the relative scratch area was measured by Image J (*P < 0.05). [file Image_2.tif]
